# The N^1^‐methyladenosine methyltransferase TRMT61A promotes bladder cancer progression and is targetable by small molecule compounds

**DOI:** 10.1002/ctm2.70137

**Published:** 2025-01-06

**Authors:** Jianjian Yin, Xin Fan, Qi Chang, Yuanheng Dai, Tao Wang, Lei Shi, Linlin Yang, Xiaoming Yang, Xudong Zhang, Lei Jin, Tao Liu, Fengmin Shao, Lirong Zhang, Dongkui Song

**Affiliations:** ^1^ Department of Pharmacology School of Basic Medical Sciences Zhengzhou University Zhengzhou China; ^2^ Department of Clinical Laboratory Henan Provincial People's Hospital People's Hospital of Zhengzhou University Zhengzhou China; ^3^ Department of Urology First Affiliated Hospital of Zhengzhou University Zhengzhou China; ^4^ Translational Research Institute Henan Provincial People's Hospital and People's Hospital of Zhengzhou University Academy of Medical Sciences Zhengzhou University Zhengzhou China; ^5^ School of Medicine and Public Health University of Newcastle Newcastle Australia; ^6^ Children's Cancer Institute Australia for Medical Research University of New South Wales Sydney Australia; ^7^ Department of Nephrology Henan Provincial Key Laboratory of Kidney Disease and Immunology Henan Provincial Clinical Research Center for Kidney Disease Henan Provincial People's Hospital Zhengzhou China

1

Dear Editor,

Bladder cancer (BLCA) is the most common malignant tumour of the urinary system and has a high recurrence rate.^1^, ^2^ N^1^‐methyladenosine (m^1^A) methylation is a key mechanism of post‐transcriptional regulation.^3^, ^4^, ^5^ m^1^A levels in the urine of BLCA patients are higher than in the urine of normal people, and TRMT61A is highly expressed in human BLCA tissues.^6^, ^7^ Although some reports are consistent with our previous results,^8^ the biological functions and potential mechanisms of action of m^1^A methylation in BLCA remain unknown.

Here, the role of TRMT61A in BLCA was assessed in vitro and in vivo. Silencing TRMT61A inhibited 5637 cell proliferation, clonogenicity, migration and invasion, while its overexpression was promoted in T24 cells (Figure S1A,B and Figure 1A–F). Total m^1^A levels in RNA decreased and increased following TRMT61A knocking‐down, and over‐expression, respectively. Quantitative ultra‐performance liquid chromatography/mass spectrometry analysis confirmed these results (Figure 1G–J). In vivo, tumour growth was effectively suppressed, as reflected by the significant reduction in tumour size and weight in the short hairpin RNA (shRNA) TRMT61A group. Immunohistochemistry staining showed a marked decrease in the proportion of tumour cells positively stained by anti‐Ki‐67 antibody in the shRNA TRMT61A group (Figure S1C and Figure 1K–M). Overall, TRMT61A promotes m^1^A modification and BLCA progression.

**FIGURE 1:**
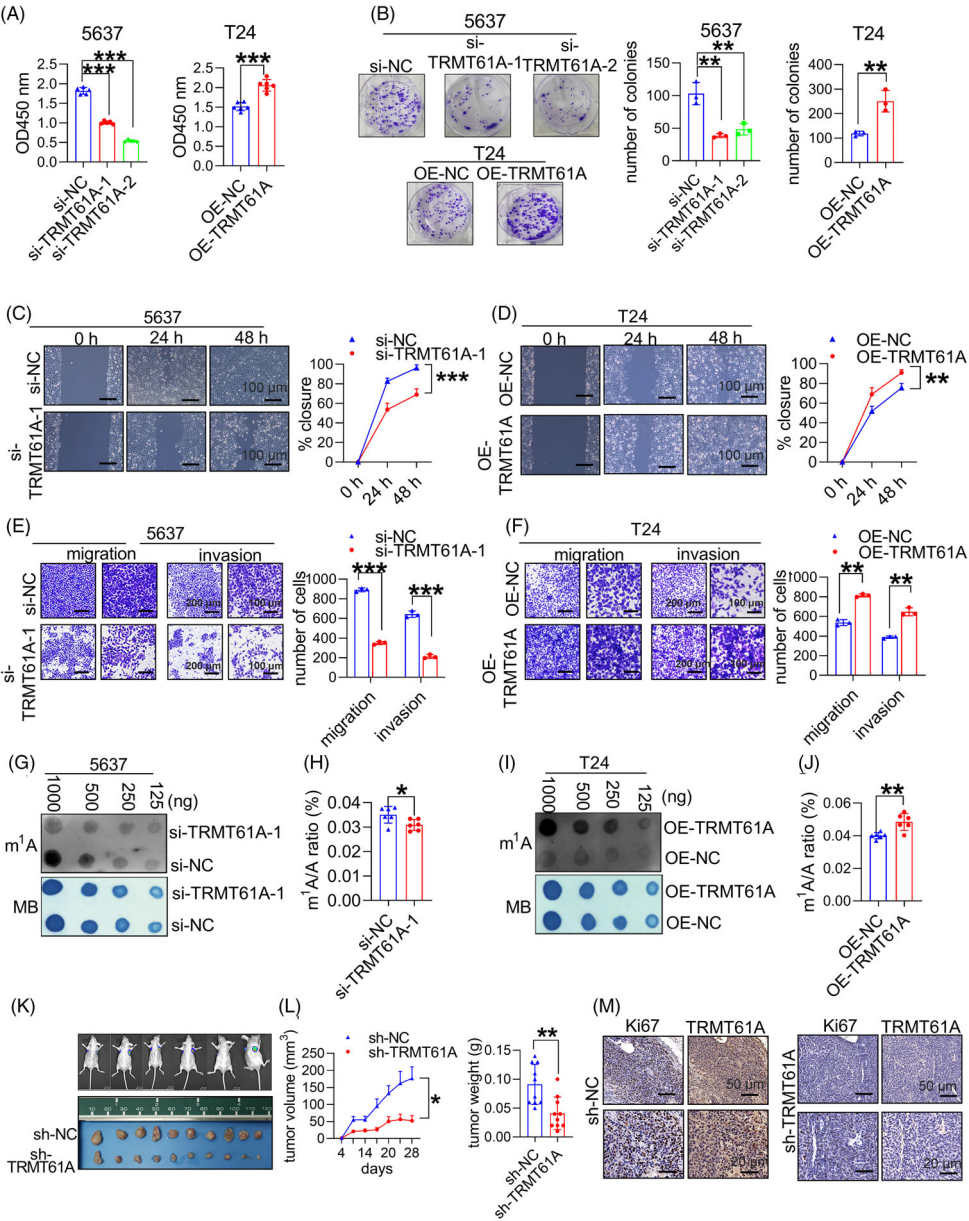
TRMT61A induces RNA N^1^‐methyladenosine (m^1^A) methylation and bladder cancer (BLCA) progression. (A, B) 5637 BLCA cells transfected with a negative control siRNA (si‐NC) or small‐interfering RNA (siRNA) targeting TRMT61A (si‐TRMT61A‐1 or siTRMT16A‐2) and T24 cells transfected with an empty vector (OE‐NC) or TRMT61A overexpression construct (OE‐TRMT61A) were subjected to Cell Counting Kit 8 cell viability (OD_450_) assays 48 h later (A) and colony formation assay 14 days later (B). Cell colonies were fixed by crystal violet, and the number of colonies was counted. (C, D) Wound healing assays were performed 48 h after siRNA or expression construct transfections; wound healing rates were measured at the indicated time points using ImageJ software. Scale bar: 100 µm. (E, F) Cell migration and invasion capabilities were measured by transwell migration and Matrigel invasion assays 48 h after siRNA or expression construct transfections; the cells were fixed with crystal violet. Scale bar: 200 µm (left, migration assays and invasion assays), 100 µm (right, migration assays and invasion assays). (G, I). The m^1^A levels in TRMT61A‐knockdown 5637 cells and TRMT61A‐overexpression T24 cells were examined by m^1^A dot blot. Corresponding RNAs from the cells were loaded equally by a 2‐fold serial dilution (1000, 500, 250 and 125 ng) for methylene blue (MB) staining as loading controls. (H, J) Ultra‐high‐performance liquid chromatography‐tandem mass spectrometry (UHPLC–MS/MS) assays were performed to detect and quantify m^1^A levels in total RNA from 5637 and T24 cells. (K) Fluorescent imaging of tumours in nude mice xenografted with 5637 cells stably transfected with a control vector (sh‐NC) or TRMT61A shRNA construct (sh‐TRMT61A). (L) Analysis of tumour volume and weight in mice xenografted with sh‐NC or sh‐TRMT61A 5637 cells, respectively. (M) Immunohistochemistry staining was performed with anti‐Ki‐67 and anti‐TRMT61A antibodies in xenograft tumours; Scale bar: 50 µm (upper panel) and 20 µm (lower panel). Two‐tailed Student's *t*‐test was used for comparisons between the two groups. Repeated measures analysis of variance (ANOVA) was used to analyze differences in tumour volume among groups at different time intervals. *p < .05, **p < .01 and *** *p *< .001.

To identify the target mRNAs of TRMT61A in BLCA, we conducted MeRIP‐seq and RNA‐seq analyses between control shRNA and TRMT61A shRNA. According to MeRIP‐seq analysis, m^1^A peaks were particularly abundant in the vicinity of 5′ untranslated regions near coding sequence regions and were found throughout the genome and across all chromosomes (Figure 2A and Figure S2A). HMOX2 mRNA was evidently m^1^A de‐methylated in the MeRIP‐seq data and downregulated in RNA‐seq data after TRMT61A knockdown, and overexpressed in human BLCA tissues; the same result was obtained with The Cancer Genome Atlas (TCGA) database (Figure 2B,C and Figure S2E). AGGCUGG/A was the top m^1^A‐modified motif; the HMOX2 mRNA m^1^A peak diminished upon TRMT61A knockdown (Figure S2F,G). Therefore, further investigations focused on HMOX2 as a TRMT61A target. We observed that HMOX2 was downregulated following TRMT61A knockdown in 5637 cells and upregulated in T24 cells overexpressing TRMT61A (Figure 2D). Using samples from BLCA patients, a positive correlation was observed between HMOX2 and TRMT61A expression; HMOX2 expression decreased in tumour tissues from mice xenografted with TRMT61A shRNA and increased in tumour tissues from BBN‐driven urinary BLCA mice (Figures S2H–K and S3). TRMT61A promoted BLCA progression by upregulating the HMOX2 level (Figure S4). Overall, TRMT61A promotes BLCA progression by upregulating HMOX2 expression. Gene Ontology and heatmap analysis revealed enrichment of genes involved in the regulation of mRNA processing and RNA splicing with decreased m^1^A methylation after TRMT61A knockdown; Kyoto Encyclopedia of Genes and Genomes pathway analysis indicated that transcripts with decreased m^1^A methylation were significantly related to the spliceosome and cancer pathways (Figure S2B–D).

**FIGURE 2:**
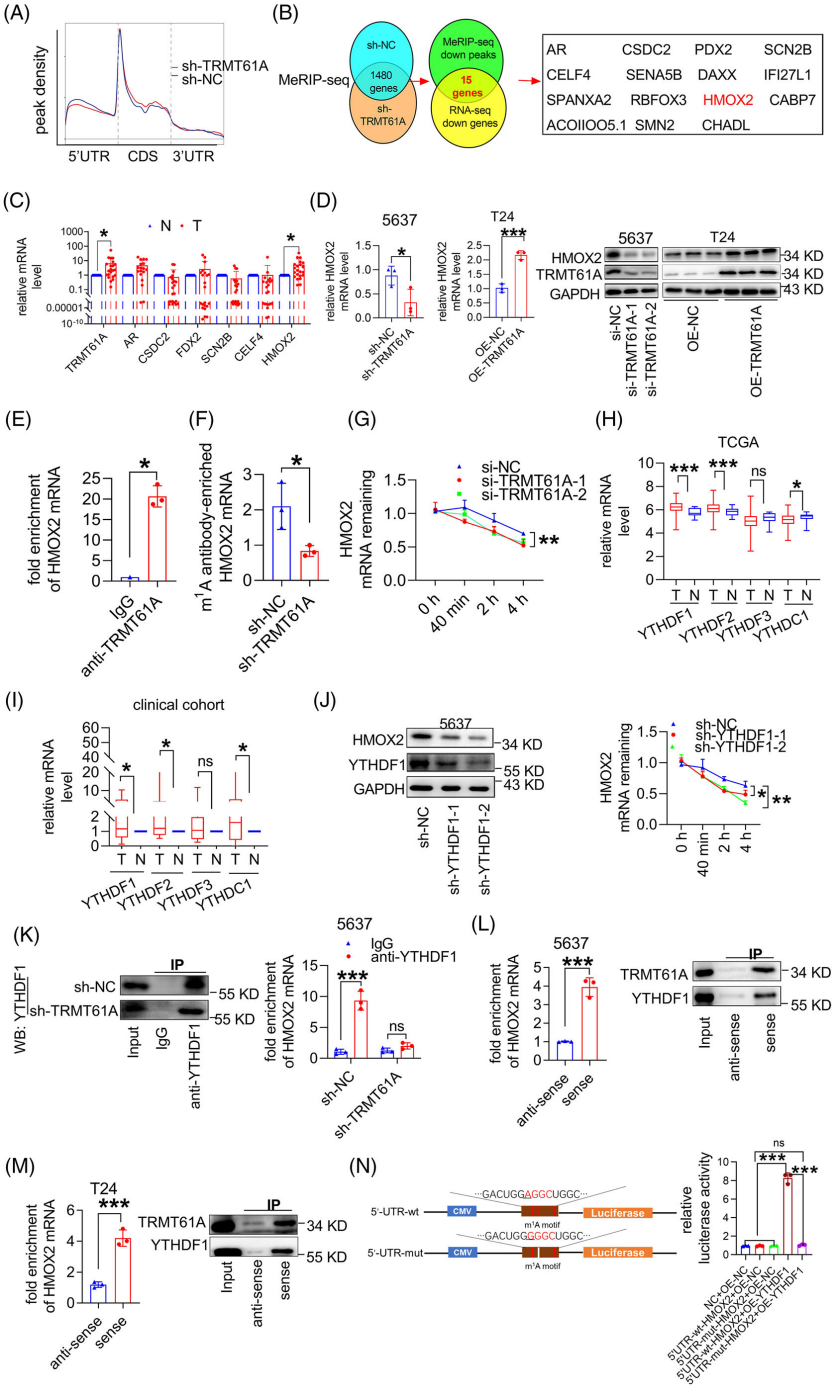
HMOX2 serves as a TRMT61A target in a YTHDF1‐dependent manner. (A) Density distribution of m^1^A peaks across mRNA transcripts. (B) Filtering of transcripts with diminished N^1^‐methyladenosine (m^1^A) peaks (MeRIP‐seq data) by transcripts with downregulated expression (RNA‐seq data) in sh‐TRMT61A, compared with sh‐NC, 5637 cells identified 15 transcripts including HMOX2 as targets of TRMT61A. (C) Expression levels of the transcripts in human BLCA tissues, and the bladder cancer tissue compared with each adjacent normal tissue (*n* = 24). T: bladder cancer tissues, and N: adjacent normal tissues. (D) Real‐time quantitative polymerase chain reaction (RT‐qPCR) and western blot analyses of HMOX2 mRNA and protein levels in 5637 cells transfected with sh‐NC or sh‐TRMT61A or in T24 cells transfected with an empty vector (OE‐NC) or TRMT61A overexpression constructs (OE‐TRMT61A). (E) RNA immunoprecipitation (RIP) assays were performed with an anti‐TRMT61A antibody or control IgG in 5637 cells, followed by RT‐qPCR analyses of HMOX2 mRNA. (F) MeRIP assays were performed with an anti‐m^1^A antibody or control IgG in sh‐NC and sh‐TRMT61A 5637 cells, followed by RT‐qPCR analysis of HMOX2 mRNA. The enrichment of m^1^A‐modified HMOX2 mRNA in each group was calculated by m^1^A‐IP/input divided by IgG‐IP/input. (G) RT‐qPCR analysis of *HMOX2* mRNA levels in 5637 cells after transfection with si‐NC or si‐TRMT61A and treatment with 5 µM actinomycin D at different time points. (H, I) Transcript levels of m^1^A modification readers (YTHDF1‐3 and YTHDC1) in human BLCA tissues and adjacent normal tissues in the TCGA database and 24 patients (in‐house clinical cohort); T: bladder cancer tissues; N: adjacent normal tissues. (J) 5637 cells transfected with control shRNA, sh‐YTHDF1‐1 or sh‐YTHDF1‐2 were treated with 5 µM actinomycin D for 40 min, 2 or 4 h, followed by RT‐qPCR analysis of HMOX2 mRNA expression. (K) The interaction between YTHDF1 protein and HMOX2 mRNA was examined by RIP assays with a control IgG or YTHDF1 antibody, followed by immunoblot analysis with a YTHDF1 antibody and RT‐qPCR analysis with primers targeting HMOX2 mRNA, in sh‐NC or sh‐TRMT61A 5637 cells. (L‐M) RNA‐binding protein pull‐down assays were performed using in vitro biotinylated anti‐sense (control) and sense (test) HMOX2 mRNAs, followed by RT‐qPCR analysis of HMOX2 mRNA and immunoblot analysis with TRMT61A and YTHDF1 antibodies in 5637 (L) and T24 (M) cells. (N) Luciferase reporter constructs were generated to express either wild‐type HMOX2 5′ UTR or HMOX2 5′ UTR with adenine mutation to guanosine in the m^1^A consensus sequences. 5637 cells were co‐transfected with luciferase reporter constructs expressing control (NC) or either wild‐type or m^1^A site mutant HMOX2 5′ UTR, together with a control vector or YTHDF1 expression construct (YTHDF1 control plasmid [OE‐NC] or YTHDF1 overexpression plasmid [OE‐YTHDF1]). Firefly luciferase activity was measured and normalized to Renilla luciferase activity. Wilcoxon's paired test was used to compare the expression of the genes in the BLCA tissue specimens and adjacent normal tissue specimens. Two‐tailed Student's *t*‐test was used for comparisons between the two groups. Repeated measures analysis of variance (ANOVA) was used to analyze mRNA stability at different time intervals for each group separately. *p < .05, **p < .01 and ***p < .001.

Mechanistically, HMOX2 mRNA was enriched by the m^1^A antibody and m^1^A modification was reduced after TRMT61A knockdown in 5637 cells. RNA immunoprecipitation (RIP) analysis confirmed the interaction between TRMT61A protein and HMOX2 mRNA in 5637 cells. TRMT61A knockdown decreased the stability of HMOX2 mRNA (Figure 2E–G). YTHDF1 was screened through human BLCA tissues and the TCGA database (Figure 2H,I). RIP assays confirmed the interaction between YTHDF1 protein and HMOX2 mRNA; the interaction was attenuated upon TRMT61A knockdown. RNA pull‐down assays confirmed that HMOX2 mRNA could directly bind to TRMT61A and YTHDF1 proteins. In mutant HMOX2, m^1^A modification was abrogated by the replacement of adenine with guanosine in the m^1^A consensus sequences. Luciferase assays revealed a significant increase in relative luciferase activity in cells transfected with the wild‐type HMOX2 construct, but there was no significant change in luciferase activity in cells transfected with the mutant HMOX2 construct (Figure 2J–N). Next, we explored why TRMT61A is abnormally overexpressed in BLCA. Eleven transcriptional regulators were identified after intersecting transcription factors from the two prediction tools; nuclear factor kappa B (NF‐κB) p56 was the transcriptional regulator most significantly associated with TRMT61A (Figure S5A). Real‐time polymerase chain reaction (RT‐PCR), western blot and IHC results showed that NF‐κB was highly expressed in cancer tissues and positively correlated with TRMT61A expression (Figure 3A–C). Subsequently, 5637 cells were treated with SN50, an NF‐κB inhibitor, which decreased p65 protein expression and phosphorylation (p‐NF‐κB, p‐p65) and reduced TRMT61A protein expression. Conversely, p‐p65 and TRMT61A levels increased after treatment with tumour necrosis factor‐alpha (TNF‐α), an NF‐κB activator, in T24 cells (Figure 3D and Figure S5B,C). However, TRMT61A knockdown or overexpression did not affect p65 and p‐p65 expression in 5637 and T24 cells (Figure S5D–F). Bioinformatics prediction by JASPAR revealed two NF‐κB binding sites; chromatin immunoprecipitation confirmed strong binding of NF‐κB at the ‐1105 to ‐1094 and ‐649 to ‐638 bp sites of the TRMT61A promoter. Experiments with dual‐luciferase reporter genes confirmed the targeting relationship (Figure 3E–G). TRMT61A lacks a catalytic nuclear localization sequence.^9^ Thus, we examined whether NF‐κB bound to TRMT61A protein and regulated its nuclear translocation. NF‐κB activation facilitated the nuclear translocation of TRMT61A (Figure 3H–L).

**FIGURE 3:**
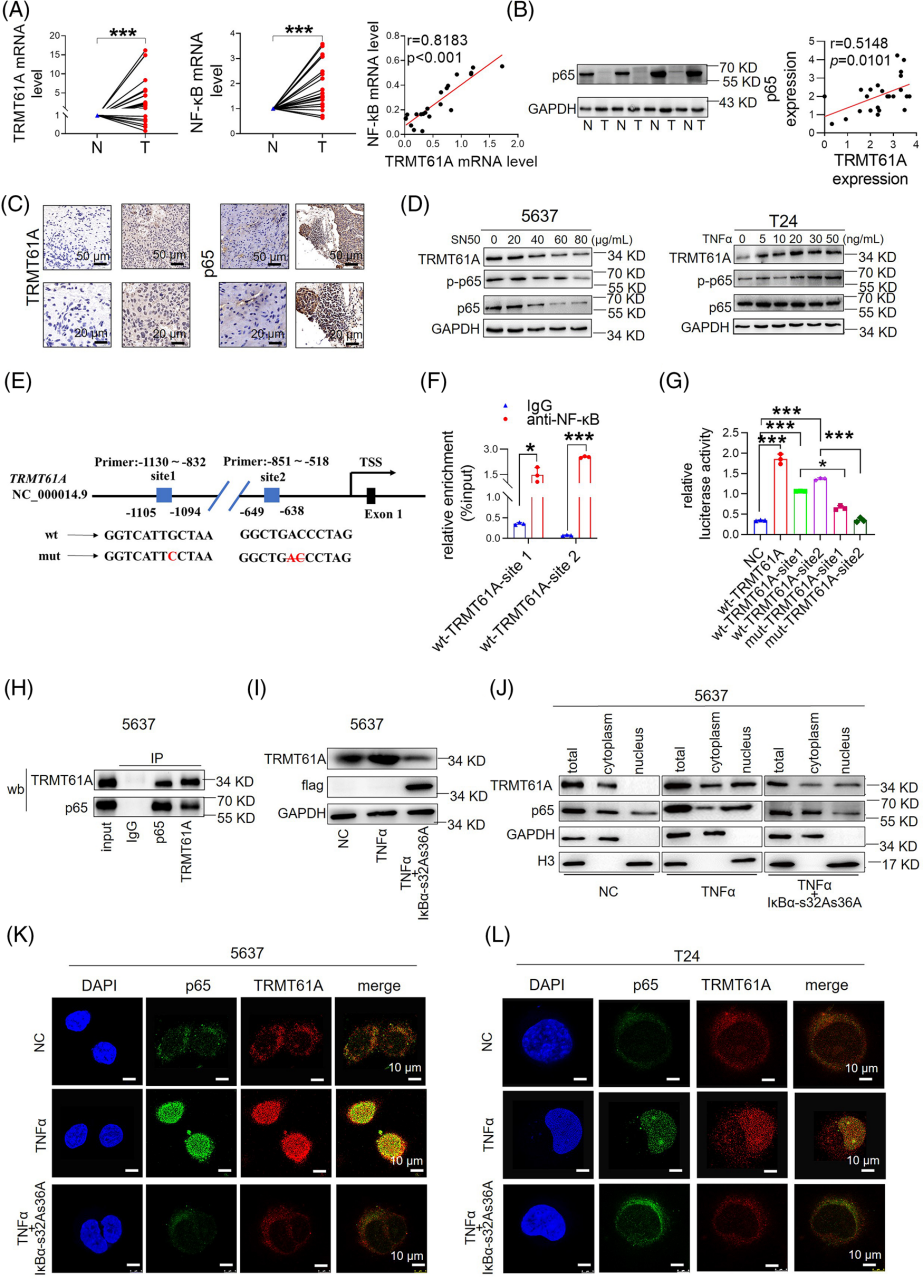
NF‐_k_B promotes TRMT61A gene transcription and TRMT61A protein nuclear translocation. (A) Transcript levels of TRMT61A and nuclear factor kappa B (NF‐κB) in bladder cancer (BLCA) (T) versus adjacent normal tissues (N) from 24 patients were examined by real‐time quantitative polymerase chain reaction (RT‐qPCR), and the correlation between TRMT61A and NF‐κB expression was examined by regression analysis. (B) Protein levels of TRMT61A and NF‐κB in BLCA (T) versus adjacent normal tissues (N) from 24 patients were examined by immunoblot, and the correlation between TRMT61A and NF‐κB expression was examined by regression analysis. (C) Immunohistochemistry (IHC) analysis of TRMT61A and NF‐κB protein expression in BLCA tissues (upper panel scale bar: 50 µm; lower panel scale bar: 20 µm). (D) 5637 cells were treated with SN50 for 24 h and T24 cells were treated with TNF‐α for 24 h, followed by western blot analysis of p65, pp65, and TRMT61A protein expression and quantification. (E) Prediction of NF‐κB binding sites at the TRMT61A promoter, genomic positions of ChIP assay primers, and mutations of the NF‐κB binding sites for the generation of wild‐type and mutant pGL3‐TRMT61A promoter constructs. (F) ChIP assays were performed with a control IgG or anti‐NF‐κB antibody and PCR with primers targeting the two NF‐κB binding sites at the TRMT61A promoter in 5637 cells. (G) 5637 cells were transfected with pGL3‐luciferase reporter constructs encoding empty vector (NC), wild type TRMT61A promoter with both of the two NF‐κB binding sites (wt‐TRMT61A), wild type TRMT61A promoter with NF‐κB binding site 1 only (wt‐TRMT61A‐site 1) or site 2 only (wt‐TRMT61A‐site 2), or mutant TRMT61A promoter with mutations at the NF‐κB binding site 1 (mut‐TRMT61A‐site 1) or site 2 (mut‐TRMT61A‐site 2) for 48 h. Firefly luciferase activity was measured and normalized to Renilla luciferase activity. (H) Co‐immunoprecipitation (Co‐IP) was performed with proteins from 5637 cells and TRMT61A or NF‐κB antibody or control IgG, followed by Western blot analysis with NF‐κB and TRMT61A antibodies. (I) Expression of TRMT61A in 5637 cells after vehicle control and/or IκBαS32AS36A treatment. (J) 5637 cells were treated with vehicle control (NC), TNF‐α and/or Flag‐tagged IκBαS32AS36A, followed by cytoplasmic and nuclear protein fractionation and immunoblot analysis of TRMT61A and p65 protein expression. GAPDH and histone H3 proteins were used as loading controls for cytoplasmic and nuclear proteins respectively. (K‐L) 5637 and T24 cells were treated with vehicle control (NC), TNF‐α and/or Flag‐tagged IκBαS32AS36A. Double immunofluorescence cytochemistry staining was performed with anti‐p65 and anti‐TRMT61A antibodies (green fluorescence represents NF‐κB/p65, and red fluorescence represents TRMT61A), followed by counterstaining of the nucleus with DAPI (blue). Wilcoxon's paired test was used to compare the expression of the genes in the BLCA tissue specimens and adjacent normal tissue specimens. Two‑tailed Student's t‑test was used to compare two groups, and a one‐way analysis of variance (ANOVA) to compare more than two groups. *p < .05 and ***p < .001.

Finally, we identified TRMT61A inhibitors using a virtual screening strategy and two compounds (abbreviated as CMP) were potentially effective in inhibiting TRMT61A (Figure 4A,B, Figures S6–S8 and Table S1). Treatment with CMP1 or CMP9 significantly reduced m^1^A levels; RIP assays with anti‐TRMT61A and anti‐m^1^A antibodies and RT‐qPCR analysis revealed that treatment with CMP1 or CMP9 significantly reduced TRMT61A protein binding to HMOX2 mRNA, HMOX2 mRNA m^1^A modification and HMOX2 mRNA expression (Figure 4C–F). In vivo, CMP1 and CMP9 showed strong anti‐tumor effects, similar to DDP and Thiram (Figure 4G,H). CMP9 showed a good safety profile, while CMP1 increased serum Urea and Crea levels, indicating kidney toxicity exerted by CMP1 (Figure 4I,J). Consistent with the results of in vitro experiments, m^1^A levels and HMOX2 mRNA expression in tumour tissues decreased after CMP1 and CMP9 treatment (Figure 4K,L). Additional studies to demonstrate the anticancer efficacy of CMP9 in different BLCA mouse models and pharmacodynamics and pharmacokinetics studies are warranted to facilitate its potential applications.

**FIGURE 4:**
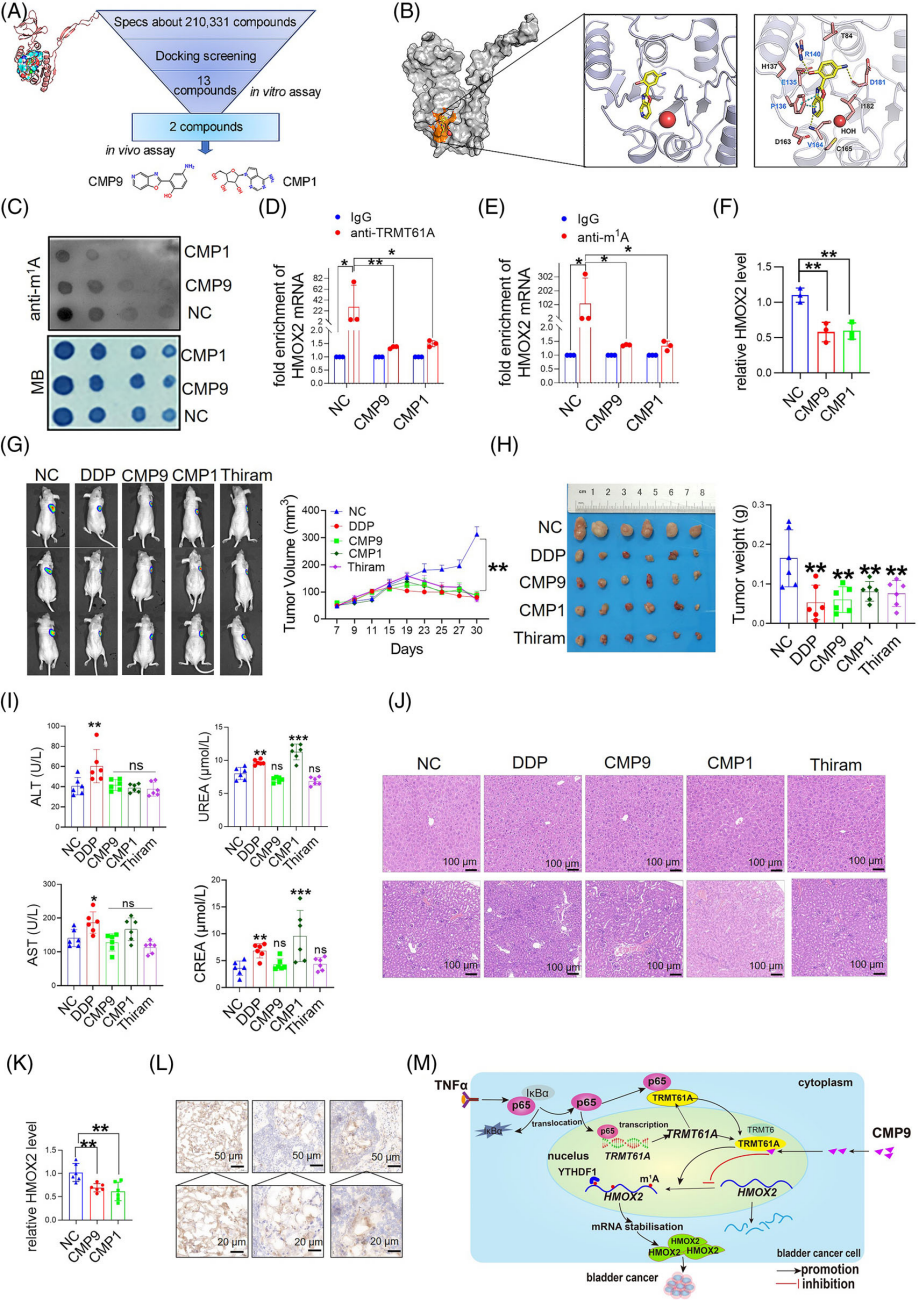
The TRMT61A inhibitor suppresses bladder cancer (BLCA) growth. (A) Workflow of the TRMT61A inhibitor discovery strategy from virtual screening to in vitro and in vivo validation. (B) The crystal structure of the TRMT61A protein was provided and molecular docking demonstrated that CMP9 is physically bound to the catalytic domain of TRMT61A. (C) 5637 cells were treated with vehicle control (NC), 0.5 µM CMP1 or 20 µM CMP9, followed by N^1^‐methyladenosine (m^1^A) dot blot assays. RNA samples from the cells were loaded by a 2‐fold serial dilution (1000 ng, 500 ng, 250 ng and 125 ng), and methylene blue staining served as the loading control. (D, E) 5637 cells were treated with vehicle control, 0.5 µM CMP1 or 20 µM CMP9. RNA immunoprecipitation (RIP) assays were performed with a control IgG, anti‐TRMT61A (D) or anti‐m^1^A (E) antibody, followed by RT‐qPCR with primers targeting HMOX2 mRNA. (F) 5637 cells were treated with vehicle control, 0.5 µM CMP1 or 20 µM CMP9, followed by RT‐qPCR analysis of HMOX2 mRNA level. (G) Nude mice were xenografted with fluorescence‐labelled 5637 cells. When tumours reached 100 mm^3^, the mice were treated with vehicle control (NC), DDP (cisplatin) (2 mg/kg for 5 days, no treatment for 2 days, and then 2 mg/kg for 5 days, i.p. injection), CMP1 (1 mg/kg for 10 days, i.p. injection), CMP9 (10 mg/kg, for 10 days, i.p. injection) or Thiram (1.6 mg/kg, for 10 days, i.p. injection) (*n* = 6 mice per group). Fluorescence imaging of xenografted tumours in live mice was taken 30 days post‐treatment (left), and tumour size was monitored during the 30 days of treatment (right). (H) Images and weights of tumours dissected from the mice after treatment with NC, DDP, CMP1, CMP9 or Thiram, when the mice were culled 30 days post‐treatment. (I) Serum ALT, AST, Urea and Crea concentrations in the mice treated with NC, DDP, CMP1, CMP9 or Thiram were examined when the mice were culled (*n* = 6 mice per group, one‐way analysis of variance [ANOVA]). (J) Representative hematoxylin‐eosin (H&E) staining of liver and kidney tissues from the mice after treatment with NC, DDP, CMP1, CMP9 or Thiram. (K) RT‐qPCR analysis of HMOX2 mRNA expression in tumours from the mice. (L) Immunohistochemical detection of m^1^A modification in tumour tissues from the nude mice treated with NC, CMP9 or CMP1, respectively; Scale bar: 50 µm (upper panel) and 20 µm (lower panel). (M) Schematic representation of TRMT61A promoting bladder cancer progression. Two‑tailed Student's *t*‑test was used to compare two groups, and one‐way ANOVA to compare more than two groups. *p < .05, **p < .01 and ***p < .001 relative to control group.

Overall, this study elucidates the molecular mechanisms via which NF‐κB increases the transcription of TRMT61A, which accelerates m^1^A modification of HMOX2 mRNA and enhances its stability via a YTHDF1‐dependent mechanism, ultimately promoting BLCA progression. The small molecule compound TRMT61A inhibitor CMP9 is a promising novel anticancer agent (Figure 4M).

## AUTHOR CONTRIBUTIONS

Jianjian Yin designed the study, performed experiments, analyzed and interpreted the data and wrote the manuscript. Dongkui Song, Lirong Zhang and Tao Liu conceived the study and reviewed and edited the manuscript. Fengmin Shao supervised the study and reviewed the manuscript. Xudong Zhang and Lei Jin supervised the experiments. Xin Fan, Qi Chang and Linlin Yang performed experiments and interpreted the data. Yuanheng Dai, Tao Wang, Lei Shi and Xiaoming Yang collected clinical specimens. All authors have read and approved the final manuscript.

## CONFLICT OF INTEREST STATEMENT

The authors declare no conflict of interest.

## FUNDING INFORMATION

This work was funded by the National Natural Science Foundation of China (Grant Nos. 82373077 and U1904162).

## ETHICS STATEMENT

This study was approved by the Ethics Committee of the First Affiliated Hospital of Zhengzhou University, and written informed consent was obtained from the patients or their relatives prior to the study. All animal experiments were approved by the Zhengzhou University Animal Care and Ethics Committee (ZZUIRB2022‐143).

## Supporting information



Supporting Information

## Data Availability

The original contributions presented in the study are included in the article/Supporting Information. The publicly available datasets analyzed in the current study are available from TCGA (https://www.cancer.gov/ccg/research/genome‐sequencing/tcga). The RNA and MeRIP sequence datasets presented in this study can be found in online repositories (GSE255629). Further information and requests for resources and reagents should be directed to and will be fulfilled by the corresponding authors.

## References

[1] Xia CF , Dong XS , Li H , et al. Cancer statistics in China and United States, 2022: profiles, trends, and determinants. Chin Med J. 2022;135:584‐590. 35143424 10.1097/CM9.0000000000002108 PMC8920425

[2] Patel VG , Oh WK , Galsky MD . treatment of muscle‐invasive and advanced bladder cancer in 2020. CA Cancer J Clin. 2020;70:404‐423. 32767764 10.3322/caac.21631

[3] Dominissini D , Nachtergaele S , Moshitch‐Moshkovitz S , et al. The dynamic N(1)‐methyladenosine methylome in eukaryotic messenger RNA. Nature. 2016;530:441‐446. 26863196 10.1038/nature16998 PMC4842015

[4] Xiong XX , Li X , Yi C . N(1)‐methyladenosine methylome in messenger RNA and non‐coding RNA. Curr Opin Chem Biol. 2018;45:179‐186. 30007213 10.1016/j.cbpa.2018.06.017

[5] Li X , Xiong X , Wang K , et al. Transcriptome‐wide mapping reveals reversible and dynamic N(1)‐methyladenosine methylome. Nat Chem Biol. 2016;12(5):311‐316. 26863410 10.1038/nchembio.2040

[6] Shi L , Yang XM , Tang DD , et al. Expression and significance of m^1^A transmethylase, hTrm6p/hTrm61p and its related gene hTrm6/hTrm61 in bladder urothelial carcinoma. Am J Cancer Res. 2015;5(7):2169‐2179. 26328247 PMC4548328

[7] Monshaugen I , Luna L , Rhodes J , et al. Depletion of the m^1^A writer TRMT6/TRMT61A reduces proliferation and resistance against cellular stress in bladder cancer. Front Oncol. 2024;13:1334112. 38304034 10.3389/fonc.2023.1334112 PMC10830773

[8] Yin JJ , Song YL , Guo YF , et al. Transcriptome‐wide 1‐methyladenosine functional profiling of messenger RNA and long non‐coding RNA in bladder cancer. Front Genet. 2024;15:1333931. 38482382 10.3389/fgene.2024.1333931 PMC10933092

[9] Macari F , El‐Houfi Y , Boldina G , et al. TRM6/61 connects PKCalpha with translational control through tRNAi(Met) stabilization: impact on tumorigenesis. Oncogene. 2016;35(14):1785‐1796. 26234676 10.1038/onc.2015.244

