# Supplementary material for: The N1‐methyladenosine methyltransferase TRMT61A promotes bladder cancer progression and is targetable by small molecule compounds
Source: Clin Transl Med. 2025 Jan 6;15(1):e70137. doi: 10.1002/ctm2.70137 (PMC11705484; doi:10.1002/ctm2.70137)
Supplement: Supplementary file 1 — Supporting Information [file CTM2-15-e70137-s001.docx]

**Supporting information**

**The N^1^-methyladenosine (m^1^A) Methyltransferase TRMT61A Promotes Bladder Cancer Progression and Is Targetable by Small Molecule Compounds**

Jianjian Yin^1, 2^, Xin Fan^3^, Qi Chang^1^, Yuanheng Dai^3^, Tao Wang^3^, Lei Shi^3^, Linlin Yang^1^, Xiaoming Yang^3^, Xudong Zhang^4, 5^, Lei Jin^5^, Tao Liu^4, 6^**^*^**, Fengmin Shao^7*^, Lirong Zhang^1^**^*^**, Dongkui Song^3^**^*^**

^1^Department of Pharmacology, School of Basic Medical Sciences, Zhengzhou University, Zhengzhou, Henan 450001, China.

^2^Department of Clinical Laboratory, Henan Provincial People's Hospital, People's Hospital of Zhengzhou University, Zhengzhou, Henan 450053, China.

^3^Department of Urology, First Affiliated Hospital of Zhengzhou University, Zhengzhou, Henan 450052, China.

^4^Translational Research Institute, People's Hospital, Academy of Medical Sciences, Zhengzhou University, Zhengzhou, Henan 450003, China.

^5^School of Medicine and Public Health, University of Newcastle, Newcastle, NSW 2308, Australia.

^6^Children's Cancer Institute Australia for Medical Research, University of New South Wales, Sydney, NSW 2308, Australia.

^7^Department of Nephrology, Henan Provincial Key Laboratory of Kidney Disease and Immunology, Henan Provincial Clinical Research Center for Kidney Disease, Henan Provincial People's Hospital, Zhengzhou, Henan 450053, China.

***Correspondence:**

Dongkui Song

[dksong@zzu.edu.cn](mailto:dksong@zzu.edu.cn)

Lirong Zhang

[lrzhang@zzu.edu.cn](mailto:lrzhang@zzu.edu.cn)

Fengmin Shao

fengminshao@126.com

Tao Liu

[tliu@ccia.unsw.edu.au](mailto:tliu@ccia.unsw.edu.au)

**Contents**

Materials and Methods

Supplementary Tables S1-7

Supplementary Figures S1-8

**Materials and Methods**

**Patients and tumor samples**

Twenty-four BLCA and adjacent tissues were collected from patients aged 45–85 years (median 68.5 years) after total or partial cystectomy at the First Affiliated Hospital of Zhengzhou University from September 2019 to September 2022 (Supplementary Table S2). Of these, eight cases and 16 patients were diagnosed with low-grade and high-grade BLCA ^[1]^, respectively. BLCA and adjacent normal tissues located 2 cm away from the cancer tissue were collected within 1 h after bladder isolation surgery. Specimens were washed with normal saline, immediately snap-frozen in liquid nitrogen within de-enzymatic cryopreservation tubes, and stored at -80 °C for later use. This study was approved by the Ethics Committee of the First Affiliated Hospital of Zhengzhou University and written informed consent was obtained from the patients or their relatives prior to the study.

**Cell culture**

Human BLCA cell lines (5637 and T24) were purchased from the Chinese Academy of Cell Resource Center (Shanghai, China). The cells were incubated in RPMI-1640 medium (Gibco, USA) containing 10% FBS (Corning, USA) and 1% penicillin and streptomycin (Solarbio, China) at 37 °C with 5% CO_2_. All cell lines were authenticated by short tandem repeat profiling and routinely tested to exclude Mycoplasma contamination.

**Animals and *in vivo* experiments**

Male immune-deficient BALB/c nude mice (4 weeks old) were purchased from Beijing Vital River Laboratory Animal Technology Co., Ltd. (Beijing, China) and male C57BL/6 mice were purchased from Beijing HFK Bioscience Co., Ltd. (Beijing, China).

In the TRMT61A knocking-down study, cells (1×10^7^) were suspended in PBS and subcutaneously implanted into the right (sh-NC‐transfected 5637 cells) and left (shTRMT61A‐transfected 5637 cells) flank of nude mice ^[2]^. In the TRMT61A small-molecule inhibitor study, 5637 cells (1×10^7^) were suspended in PBS and subcutaneously implanted. When tumor volume reached 100 mm^3^, the tumor-bearing mice were randomly distributed into different treatment groups. Tumor volume was recorded using an IVIS Spectrum animal imaging system (PerkinElmer, USA), which detected tumor growth with 100 μL of D-luciferin potassium salt (15 mg/mL; MeilunBio, China) per mouse.

In BBN-induced BLCA mouse model study, BLCA was induced in C57BL/6 mice by administering 0.05% N-butyl-N-(4-hydroxybutyl) nitrosamine (BBN, Macklin) in drinking water for 25 weeks.

Nude mice were xenografted with fluorescence-labelled 5637 cells. When tumors reached 100 mm3, the mice were treated with vehicle control (NC), DDP (cisplatin) (2 mg/kg for 5 days, no treatment for 2 days, and then 2 mg/kg for 5 days, i.p. injection), CMP1 (1 mg/kg for 10 days, i.p. injection), CMP9 (10 mg/kg, for 10 days, i.p. injection), or Thiram (1.6 mg/kg, for 10 days, i.p. injection) (n=6 mice per group). Fluorescence imaging of xenografted tumors in live mice was taken 30 days post-treatment, and tumor size was monitored during the 30 days of treatment.

Finally, the mice were sacrificed by cervical dislocation, tumors were weighed, tumor volumes were measured, and tumors were collected for further immunohistochemistry (IHC) staining, RT-qPCR, western blotting (primer information can be found in Table S3). All animal experiments were approved by the ZhengZhou University Animal Care and Ethics Committee (ZZUIRB2022-143).

**RNA sequencing (RNA-Seq) and methylated RNA immunoprecipitation sequencing (MeRIP‑Seq)**

CloudSeq Biotech (Shanghai, China) performed RNA-seq and MeRIP-seq sample sequencing. For RNA-seq, total RNA was extracted and rRNA removed following the manufacturer's instructions. Then RNA libraries were constructed. Libraries were controlled for quality and quantified using the BioAnalyzer 2100 system (Agilent Technologies, USA). Library sequencing was performed on an Illumina Novaseq 6000 instrument with 150 bp paired-end reads. For MeRIP‑seq, fragmented RNA was incubated with an anti-m^1^A polyclonal antibody in immunoprecipitation (IPP) buffer for 2 h at 4 °C, and it was then immunoprecipitated with protein A magnetic beads at 4 °C for 2 h. The bound RNA was eluted from the beads with the m^1^A antibody in IPP buffer and extracted using TRIzol reagent. Both the input (without immunoprecipitation) and the m^1^A-immunoprecipitated samples were subjected to 150 bp paired-end sequencing on an Illumina HiSeq sequencer (Illumina, San Diego, CA, USA) by CloudSeq Biotech.

**The detection of m^1^A levels was performed using ultra-high performance liquid chromatography (UHPLC)-MS/MS**

According to the experimental method established in the early stage of our research ^[3]^, the operation process involved loading the internal standard through the solid-phase extraction column and collecting the effluent. UPLC measures the concentration of nucleosides in cells using an internal standard method for quantification and MRM mode measurements. the peak time of the unknown peak of the tested sample was compared with that of the nucleoside standard to identify a variety of nucleosides. The ratio of different concentrations of the nucleoside standard to the internal standard was used to establish a working curve, which was used to calculate nucleoside concentrations.

**Cell proliferation****, migration, and invasion assays**

CCK8 was performed to assess cell viability and wound healing, Transwell, and Matrigel assays were used to determine cell motility/invasion. To investigate cell proliferation, we performed a colony formation assay. After preparing single-cell suspensions, 2000 cells/well were seeded into a 6-well plate. After 11–14 days, cells were fixed in absolute ethyl alcohol and stained with 0.4% crystal violet (Solarbio, Beijing, China). After three immersion washes in pure water, each plate was scanned using an inverted microscope (Olympus, Tokyo, Japan).

**RNA extraction and reverse transcription-quantitative polymerase chain reaction (RT-qPCR)**

Total RNA from BLCA tissues or cell lines was isolated using TRIzol reagent (Thermo Fisher Scientific, Waltham, MA, USA) and quantified using a NanoDrop 2000 spectrometer (Thermo Fisher). cDNA was reverse-transcribed from 1 μg of total RNA using the Prime Script RT Reagent kit (Takara Bio, Japan) according to the manufacturer’s instructions and then used as a template for qPCR using the SYBR Green Master Mix kit (Thermo Fisher). The thermal cycling program was as follows: 50 °C for 2 min, denaturation at 95 °C for 2 min, followed by 40 cycles at 95 °C for 15 sec and 60 °C for 1 min. The primers were synthesized by Beijing Liuhe Huada Gene Technology Co. (Supplementary Table S3).

**Western blot**

Western blotting was performed as described previously ^[4]^ using the primary and secondary antibodies listed in Supplementary Table S4. The results were quantitatively analyzed using the AlphaView software (ProteinSimple, Santa Clara, CA, USA).

**Cell transfection**

Specific shRNAs and siRNAs were synthesized by GenePharma or GeneChem Technology (Shanghai, China). HMOX2, YTHDF1, and NF-κB plasmids were purchased from iGeneBio (Guangzhou, China) or GenePharma. PCDNA3.1(+)-TRMT61A plasmid was generated by cloning the full-length open reading frame of the human TRMT61A gene (NM_152307). Stable KD of TRMT61A was generated with the “pHBLV-U6-MCS-EF1-luc-T2A-Puro” vector synthesized by Hanbio Biotechnology (Shanghai, China). For the construction of stable cell lines (sh-TRMT61A), 293T packaging cell lines were used for lentiviral amplification. Lentiviral infection was carried out according to the Hanbio’s instructions. Transfection of shRNA/siRNA or plasmids was performed using Lipofectamine 3000 (Invitrogen, USA), following the manufacturer’s instructions. The oligonucleotide sequences are listed in Supplementary Table S5.

**Dot blot assays**

Detailed experimental protocols can be found in the previously published literature ^[5,6]^. Briefly, purified RNA was denatured and spotted onto a Magna nylon transfer membrane (GE Healthcare, Chicago, IL, USA), followed by UV-crosslinking. The membrane was washed with Tris-buffered saline with Tween 20 (PBST), incubated with 5% skim milk, and then incubated with an anti-m^1^A antibody for 1 h at room temperature. Following three washes with PBST, the membranes were further incubated with goat anti-mouse IgG-HRP for 1 h at room temperature, washed four times with PBST (Supplementary Table S4), and visualized using High-sig ECL Western Blotting Substrate (Amersham ImageQuant 800, Japan). Then, 0.02% methylene blue in 0.3 M sodium acetate was used to visualize the total RNA.

**Immunofluorescence cytochemistry**

Cells were seeded on a confocal dish and pretreated with TNF-α (30 ng/mL; MedChemExpress) or Flag-tagged IκBα-S32AS36A (a non-degradable mutant of IκBα). The primary antibodies, including anti-rabbit TRMT61A antibody (1:200; Invitrogen) and anti-mouse NF-κB antibody (1:200; Cell Signaling Technology, Danvers, MA, USA), were incubated overnight at 4°C, followed by incubation with secondary antibodies conjugated to Alexa Fluor 488 (1:200; Abcam, Cambridge, UK) or Alexa Fluor 594 (1:200; Abcam) at 37°C for 1 h. Finally, nuclei staining was performed using DAPI. Immunofluorescence staining was performed using an FV10 confocal laser-scanning microscope (Olympus).

**Immunohistochemistry (IHC)**

IHC assays were performed as described previously ^[7]^ using the primary antibodies including the rabbit anti-TRMT61A antibody, rabbit anti-Ki67 antibody, rabbit anti-NF-κB，rabbit anti-m^1^A and secondary anti-rabbit antibody (Supplementary Table S4). The slides were imaged and visualized under a microscope (Olympus).

**RNA stability assay**

The 5637 cells were treated with 5 μg/mL actinomycin D (MedChemExpress, USA) to suppress global mRNA transcription. Cells were harvested at 0, 45 min, 2, and 4 h after treatment, and total RNA was extracted for reverse transcription. The levels of *HMOX2* mRNA after transcriptional inhibition were determined using qRT-PCR.

**RNA pull-down assay**

Based on the manufacturer’s instructions, a magnetic RNA–protein pull-down kit (Thermo Fisher, USA) was used for *in vitro* RNA pull-down. The RNA probes of *HMOX2* were incubated with streptavidin-modified magnetic beads (Biolinkedin, China) before the cell lysates were added. The RNA–protein IP complexes were then washed three times before the immunoprecipitated protein results were analyzed using western blotting.

**RNA immunoprecipitation (RIP) assay**

The RIP assay was performed using the Magna RIP kit (Millipore), according to the manufacturer’s instructions. Briefly, anti-TRMT61A (Thermo Fisher), anti-YTHDF1 (Proteintech, China), anti-m1A (MBL), and anti-IgG (Millipore) antibodies were incubated with 25 μL magnetic beads before cell lysates were added. The RNA–protein IP complexes were then washed thrice. Finally, the immunoprecipitated RNA was analyzed using RT-qPCR.

**Dual-luciferase reporter assay**

DNA fragments of the TRMT61A promoter regions (-1105 to -1094, and -649 to -638 bp) either separately or together (-1105 to -638) and the 5′-UTR of HMOX2 containing the wild-type m^1^A and mutant (A was replaced by G) motifs were cloned from 5637 cells into the pGL-3 vector. Transfections were performed using Lipofectamine 3000 (Thermo Fisher), following the manufacturer’s protocols. The cloning and mutagenesis primers used were listed in Supplementary Table S6. All cells were harvested 48 h post-transfection and analyzed using a dual-luciferase reporter gene assay system (Promega, USA). The ratio of firefly luciferase activity to Renilla luciferase activity (as a control) was calculated.

**Chromatin immunoprecipitation (ChIP) assay**

ChIP assays were performed as described previously ^[8]^. Cell lysates were sonicated with a Bioruptor® Pico sonication system (Diagenode, Belgium) to obtain DNA fragments and immunoprecipitated overnight at 4 °C using an anti-NF-κB antibody or control IgG. DNA samples were purified using a PCR purification kit (QIAGEN, China), followed by qPCR analysis with specific primers (Supplementary Table S7) using the SYBR Green method, and enrichment was calculated as the percentage of chromatin input.

**Coimmunoprecipitation (Co-IP)**

Co-IP was performed on 5637 cells. The total protein sample was collected, lysed using RIPA buffer, and incubated with NF-κB and TRMT61A antibodies overnight at 4°C. Dynabeads Protein A/G (20 μL; Millipore) were added to the sample solution and incubated for 2 h at 4°C, followed by three washes with cold PBS. A 2× loading buffer was used to elute the sample, which was then heated at 100°C for 10 min and cooled to room temperature for western blot analysis.

**Virtual screening for small molecule compounds targeting TRMT61A**

Molecular docking screening was performed using AutoDock Vina. The crystal structure of the TRMT61A was obtained from the Protein Data Bank (PDB, 5CCB) for the docking studies. Open Babel (version 3.0.0) was used for ligand energy minimization, and Biopython was used for protein preparation ^[9,10]^. We screened the library from the Specs databases ([www.Specs.net](http://www.Specs.net)), containing approximately 210,331 commercially available compounds. Receptors and databases were prepared by AutoDockTools. The protonated states of their ionizable side chains were determined based on the most probable states of the amino acid residues Asp, Glu, Lys, Arg, and His at pH 7. After docking, the optimal conformations of each ligand were found and the optimal configuration scores of the ligands were ordered to find the compound with the highest score. Finally, 13 compounds from Specs were purchased (Supplementary Table S1).

**Statistical analysis**

Statistical analyses were performed using GraphPad Prism 9.0 (GraphPad, USA) and SPSS 21.0 software (SPSS Inc, USA). The experiments were independently repeated at least three times, and representative data were presented as the mean ± SD or SEM. Quantitative data were compared using a two-tailed Student’s *t*-test, whereas qualitative data were evaluated using the chi-square test. One-way analysis of variance (ANOVA) followed by Tukey's post-hoc test was used to analyze differences among more than two groups. A two-tailed Student's *t*-test was used for comparisons between two groups. Wilcoxon's paired test was used to compare the expression of gens in the BLCA tissue specimens and adjacent normal tissues specimens. Repeated measures ANOVA was used to analyze wound closure at different time intervals for each group. Correlation analysis of gene expression was conducted using linear regression. *P*-values for every result are labeled in the figures, and *P*<0.05 was considered statistically significant.

Supplementary Table S1.

**Supplementary Table S1.** **Small molecule compound TRMT61A inhibitors**

| No | Specs ID | docking score | Molecular Weight |
| --- | --- | --- | --- |
| CMP1 | AL-398/40897046 | -9.842 | 266.26 |
| CMP 2 | AN-329/40036830 | -7.989 | 332.38 |
| CMP 3 | AE-848/20954001 | -8.669 | 308.29 |
| CMP 4 | AO-623/37372024 | -8.733 | 335.3 |
| CMP 5 | AO-476/43421025 | -8.61 | 440.51 |
| CMP 6 | AO-476/43421028 | -9.593 | 342.38 |
| CMP 7 | AF-399/15539354 | -8.775 | 393.44 |
| CMP 8 | AB-131/42301750 | -8.554 | 386.36 |
| CMP 9 | AP-970/43483014 | -8.594 | 227.22 |
| CMP 10 | AN-988/37365012 | -7.726 | 283.31 |
| CMP 11 | AN-979/41971667 | -8.596 | 352.34 |
| CMP 12 | AO-022/43512352 | -7.938 | 352.41 |
| CMP 13 | AQ-750/42051383 | -8.229 | 333.4 |

Supplementary Table S2.

**Supplementary Table S2. The characteristics of BLCA patients**

| Parameters | Number of cases |
| --- | --- |
| Patients (n) | 24 |
| Age median (range) | 68.5 years (45-85) |
| Sex | Male=20  Female=4 |
| Stage | pTa-pT1*=4  pT2-pT4*=20 |
| Grade | High=16  Low=8 |

*pTa = low-grade non-muscle invasive bladder cancer; pT1 = intermediate risk non-muscle invasive bladder cancer; pTis = in situ neoplasia; pT2 = tumor invades muscle; pT3 = tumor invades perivesical tissue; pT4 = tumor invades prostate, uterus or vagina and pelvic wall or abdominal wall.

Supplementary Table S3.

**Supplementary Table S3.** **Oligonucleotide sequences for RT-qPCR primers**

| Gene | Forward (5′-3′) | Reverse (5′-3′) |
| --- | --- | --- |
| GAPDH | GACCTGACCTGCCGCCTA | AGGAGTGGGTGTCGCTGT |
| TRMT61A | CCAGTCAGGTTCAACATGGAAG | TGTGCGTCACCCAGTTCA |
| NF-кB | GTGGCAGTCTTCTCAAAGC | TCCAGGTCATAGAGAGGCTCCA |
| HMOX2 | TTAACCGGATGCTACGGGTG | TGACTCGTCTACCCCCTCTG |
| Me-RIP-HMOX2 | TGGGGAAAGCCACATACTCCG | GCCAGCCTCCAGTCAGCGT |
| YTHDF1 | ATGTCGGCCACCAGCGTGGACA | TCATTGTTTGTTTCGACTCTGC |
| YTHDF2 | TAGCCAACTGCGACACATTC | CACGACCTTGACGTTCCTTT |
| YTHDF3 | TGACAACAAACCGGTTACCA | TGTTTCTATTTCTCTCCCTACGC |
| YTHDC1 | GGAGGGCCAAATCTCCTACG | CTTTTCGGACAGCACGAACG |

Supplementary Table S4.

**Supplementary Table S4.** **Antibody Information**

| Antibody | Dilutions | Supplier | Catalog Number |
| --- | --- | --- | --- |
| Mouse anti-GAPDH | 1:20000 | Proteintech | 60004-1-Ig |
| Mouse anti-NF-κb (p65) | 1:1000 | Cell Signaling Technology | #6956 |
| Mouse anti-p-NF-κB | 1:1000 | ThermoFisher | 33-9900 |
| Rabbit anti-TRMT61A | 1:1000 | ThermoFisher | PA5-76553 |
| Rabbit anti-HMOX2 | 1:800 | Proteintech | 14817-1-AP |
| Rabbit anti-YTHDF1 | 1:2000 | Proteintech | 17479-1-AP |
| Rabbit anti-IκBα | 1:2000 | Abcam | Ab32518 |
| Mouse anti-m^1^A | 1:500 | MBL | D345-3 |
| Mouse anti-Flag | 1:10000 | Proteintech | 66008-4-Ig |
| Rabbit anti-Histone-H3 | 1:2000 | Abcam | Ab176842 |
| Rabbit anti-IgG | 1:200 | Millipore | 12-370 |
| HRP-conjugated Affinipure Goat Anti-Rabbit IgG(H+L) | 1:10000 | Proteintech | SA00001-2 |
| HRP-conjugated Affinipure Goat Anti-Mouse IgG(H+L) | 1:10000 | Proteintech | SA00001-1 |

Supplementary Table S5.

**Supplementary** **Table S5.** **Oligonucleotide sequences of siRNAs and shRNAs**

| Gene | Forward (5′-3′) | Reverse (5′-3′) |
| --- | --- | --- |
| TRMT61A (for cloning) | GGCACTCAGTTGACCTTATCGG | AGACCTGTGGCAGCACCTCC |
| si- TRMT61A-1 | GGCACUCAGUUGACCUUAUTT | AUAAGGUCAACUGAGUGCCTT |
| si- TRMT61A-2 | CAUACGAGGAGCUGAUCAATT | UUGAUCAGCUCCUCGUAUGTT |
| si-NC | UUCUCCGAACGUGUCACGUTT | ACGUGACACGUUCGGAGAATT |
| sh-TRMT61A | GATCCGGCACTCAGTTGACCTTATTTCAAGAGAATAAGGTCAACTGAGTGCCTTTTTTG | AATTCAAAAAAGGCACTCAGTTGACCTTATTCTCTTGAAATAAGGTCAACTGAGTGCCG |
| sh-HMOX2-1 | CCGGCATGCGTAAATGCCCTTTCTACTCGAGTAGAAAGGGCATTTACGCATGTTTTTG | AATTCAAAAACATGCGTAAATGCCCTTTCTACTCGAGTAGAAAGGGCATTTACGCATG |
| sh-HMOX2-2 | CCGGCAGTTCTACCTGTTTGAGAATCTCGAGATTCTCAAACAGGTAGAACTGTTTTTG | AATTCAAAAACAGTTCTACCTGTTTGAGAATCTCGAGATTCTCAAACAGGTAGAACTG |
| sh-YTHDF1-1 | CACCGTCCACCCATAAAGCATAACATTTCAAGAGAATGTTATGCTTTATGGGTGGATTTTTTG | GATCCAAAAAATCCACCCATAAAGCATAACATTCTCTTGAAATGTTATGCTTTATGGGTGGAC |
| sh-YTHDF1-2 | CACCGCAGGCTGGAGAATAACGACAATTCAAGAGATTGTCGTTATTCTCCAGCCTGTTTTTTG | GATCCAAAAAACAGGCTGGAGAATAACGACAATCTCTTGAATTGTCGTTATTCTCCAGCCTGC |

Supplementary Table S6.

**Supplementary Table S6.** **Oligonucleotide sequences for cloning and mutagenesis**

| Gene | Forward (5′-3′) | Reverse (5′-3′) |
| --- | --- | --- |
| TRMT61A | TAAGGTACCGGCCCTTGCCAGACATTAGC | TAACTCGAGGCAGGCATCGCTGACCAT |
| wt-TRMT61A-site1 | TTGGTACCGCTGCTCACCAGACCCAA | TTCTCGAGGGACACTTCGCCATAGACATT |
| wt-TRMT61A-site2 | TTGGTACCTGAGTGACTGGGGAGGGT | TTCTCGAGCTTTTGGGTCTGGTGAGC |
| mut-TRMT61A-site1 | AGCTCTCCCAAGCCTTTTAGAACCGCCCCTTGA | AAAAGGCTTGGGAGAGCTCTGTGCAGCAGATGG |
| mut-TRMT61A-site2 | CCCTAGCTGGCTTGGGTTCGGGCGCAGGCTAAA | AACCCAAGCCAGCTAGGGCACCCTCCCCAGTCA |
| wt-HMOX2 | CCTGAGGGAGTCGCTGACGG | GAAGCCGGGCGGAAGGAA |
| mut-HMOX2 | ACGCTGACTGGgGGCTGGCGGACAGGCGACAG | CAGCCcCCAGTCAGCGTGCCCGTCAGCGACTC |

Supplementary Table S7.

**Supplementary** **Table S7.** **Oligonucleotide sequences for ChIP PCR primers**

| Gene | Forward (5′-3′) | Reverse (5′-3′) |
| --- | --- | --- |
| TRMT61A site 1 | GCTGCTCACCAGACCCAA | GGACACTTCGCCATAGACATT |
| TRMT61A site 2 | CCAGAGGCGAGAGAGTTGAGAGTGC | AGAACAGGGAAGCGGGAG |

Supplementary Figure S1.


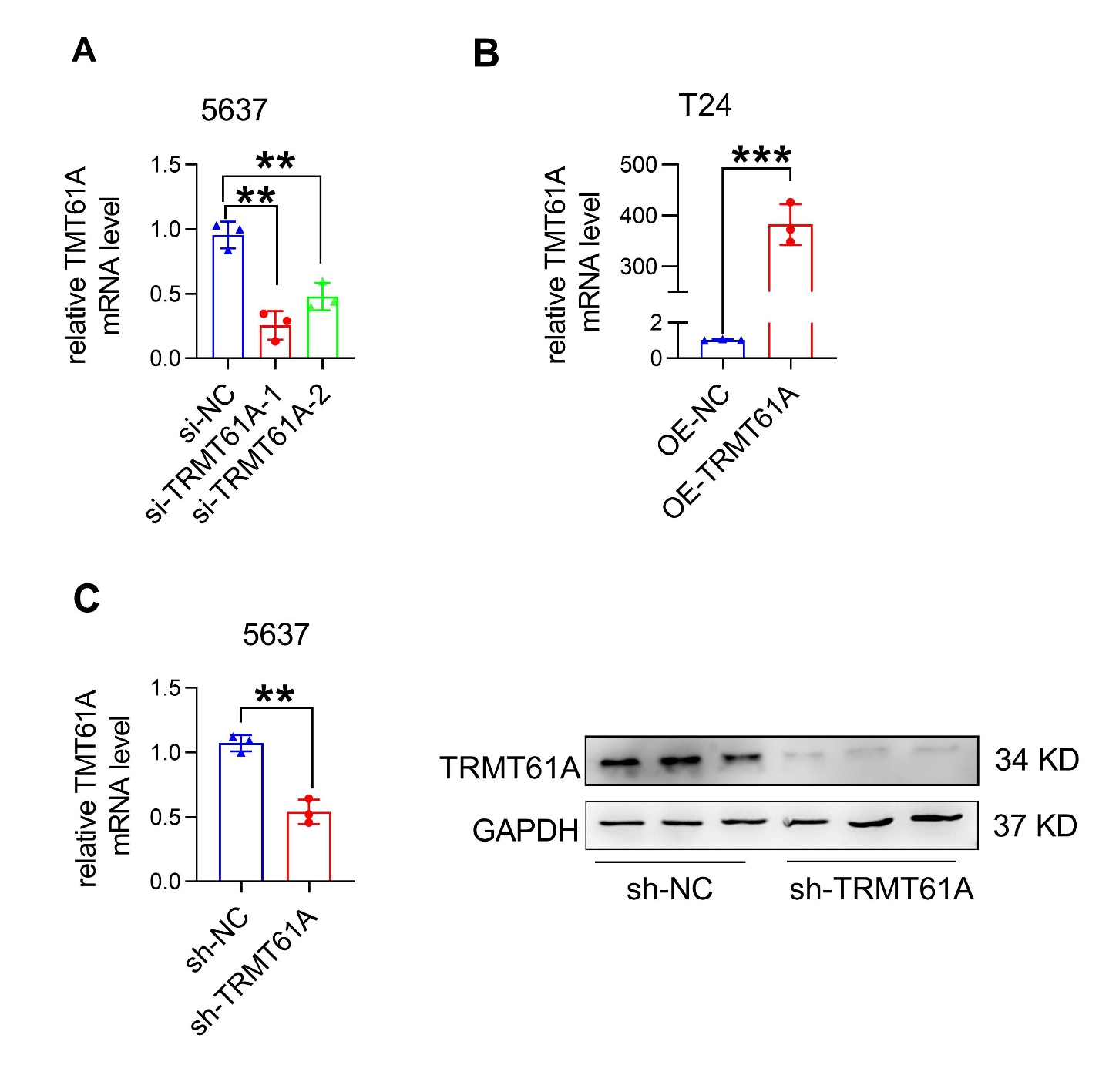


**Supplementary Figure S1.** **Transfection efficiency was verified by** **RT-qPCR and western blot.** (**A**) 5637 cells were transfected with a control siRNA (si-NC) or siRNA targeting TRMT61A (si-TRMT61A) and **(B)** T24 cells were transfected with an empty vector (OE-NC) or TRMT61A overexpression (OE-TRMT61A) construct. RT-qPCR was performed to demonstrate that the cells were transfected efficiently. (**C**) 5637 cells were stably transfected with negative control shRNA (sh-NC) or TRMT61A shRNA (sh-TRMT61A) construct. TRMT61A knockdown by shRNA was confirmed by RT-qPCR and western blot. Two‑tailed Student’s t‑test was used to compare two groups, and one-way ANOVA to compare more than two groups.

Supplementary Figure S2.


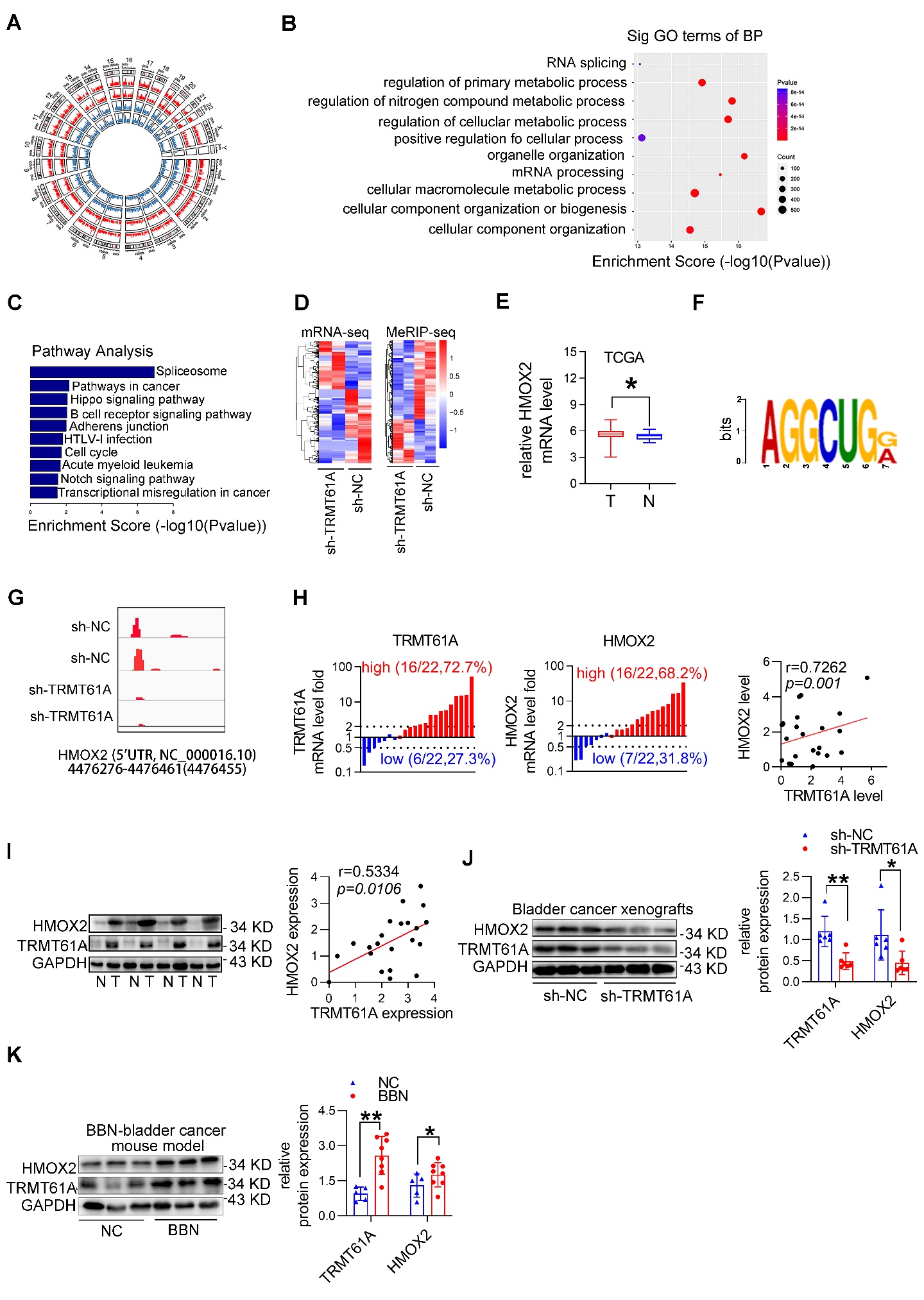


**Supplementary Figure S2.** **HMOX2 is a downstream target of TRMT61A.** (**A**) MeRIP-Seq was performed with a control IgG or anti-m^1^A antibody in sh-NC and sh-TRMT61A 5637 cells. Circos plot showing the distribution of m^1^A methylation sites on each chromosome. Blue represents sh-TRMT61A cells, and red represents sh-NC 5637 cells. (**B)** Top 10 GO terms for biological processes significantly enriched for transcripts with decreased m^1^A methylation were identified by m^1^A MeRIP-Seq in BLCA cells after TRMT61A knockdown. (**C**) KEGG enrichment analysis of m^1^A MeRIP-Seq data identified pathways correlated with m^1^A modification reduction in 5637 cells after TRMT61A knockdown. (**D**) Heatmaps of RNA-Seq and MeRIP-Seq analyses showing differentially expressed or m^1^A-modified transcripts in 5637 cells with or without *TRMT61A* knockdown. (**E**) The expression of HMOX2 in BLCA tumor tissues (T), compared with that in normal tissues (N), from the TCGA database. (**F-G**) MeRIP-Seq data showed the diagram of the motif (AGGCU) with m^1^A modification. And diminished m^1^A modification of HMOX2 mRNA in 5637 cells with TRMT61A knockdown (sh-TRMT61A). (**H**) Transcript levels of TRMT61A and HMOX2 in BLCA versus adjacent normal tissues (n=24) from patients, and regression analysis of the relationship between TRMT61A and HMOX2 mRNA levels. (**I**) Western blot analysis of TRMT61A and HMOX2 protein expression in the 24 BLCA (T) versus adjacent normal tissues (N), and analysis of the correlation between TRMT61A and HMOX2 protein expression. (**J**) Western blot analysis of TRMT61A and HMOX2 protein levels in tumor tissues from nude mice xenografted with sh-NC or sh-TRMT61A 5637 cells (n=6). (**K**) HMOX2 and TRMT61A protein levels in tumor tissues from BBN-driven urinary BLCA mice and bladder tissues from un-treated control (NC) mice were examined by western blot (n=5-8). GAPDH was used as the loading control. Two-tailed Student’s *t*-test was used for comparisons between two groups. Wilcoxon's paired test was used to compare the expression of gens in the BLCA tissue specimens and adjacent normal tissues specimens. **P*<0.05, ***P*<0.01, and ****P*<0.001.

Supplementary Figure S3.


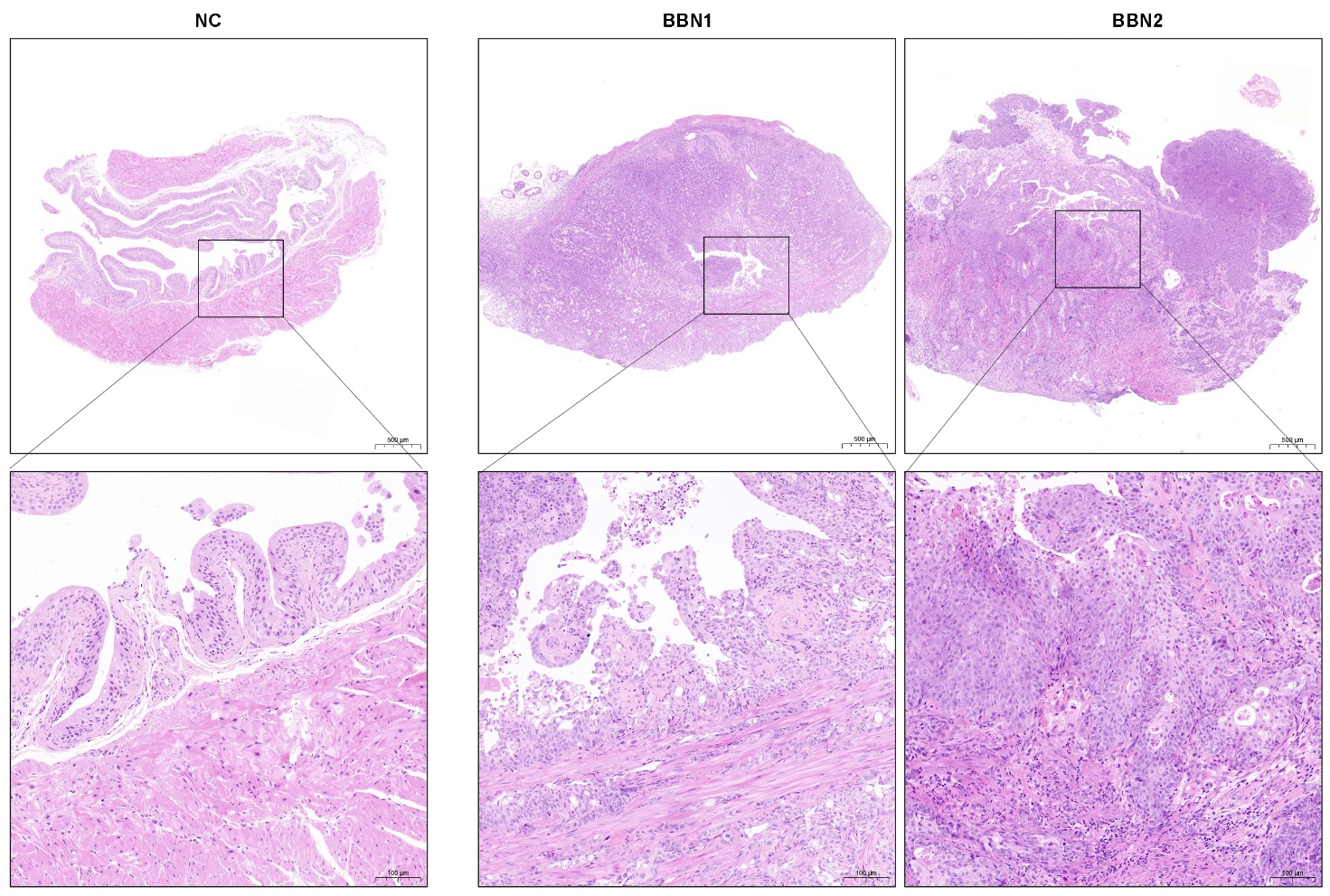


**Supplementary Figure S3.** **Representative hematoxylin eosin (H&E) staining of normal bladder and BBN-induced bladder tumor tissues.** NC group received normal drinking water throughout 25 weeks. BBN group mice received 0.05% of N-butyl-N-(4-hydroxybutyl)-nitrosamine (BBN) in the drinking water for 25 weeks. Scale bar = 200 μm for the upper row; and scale bar = 100 μm for the lower row; n=6.

Supplementary Figure S4.


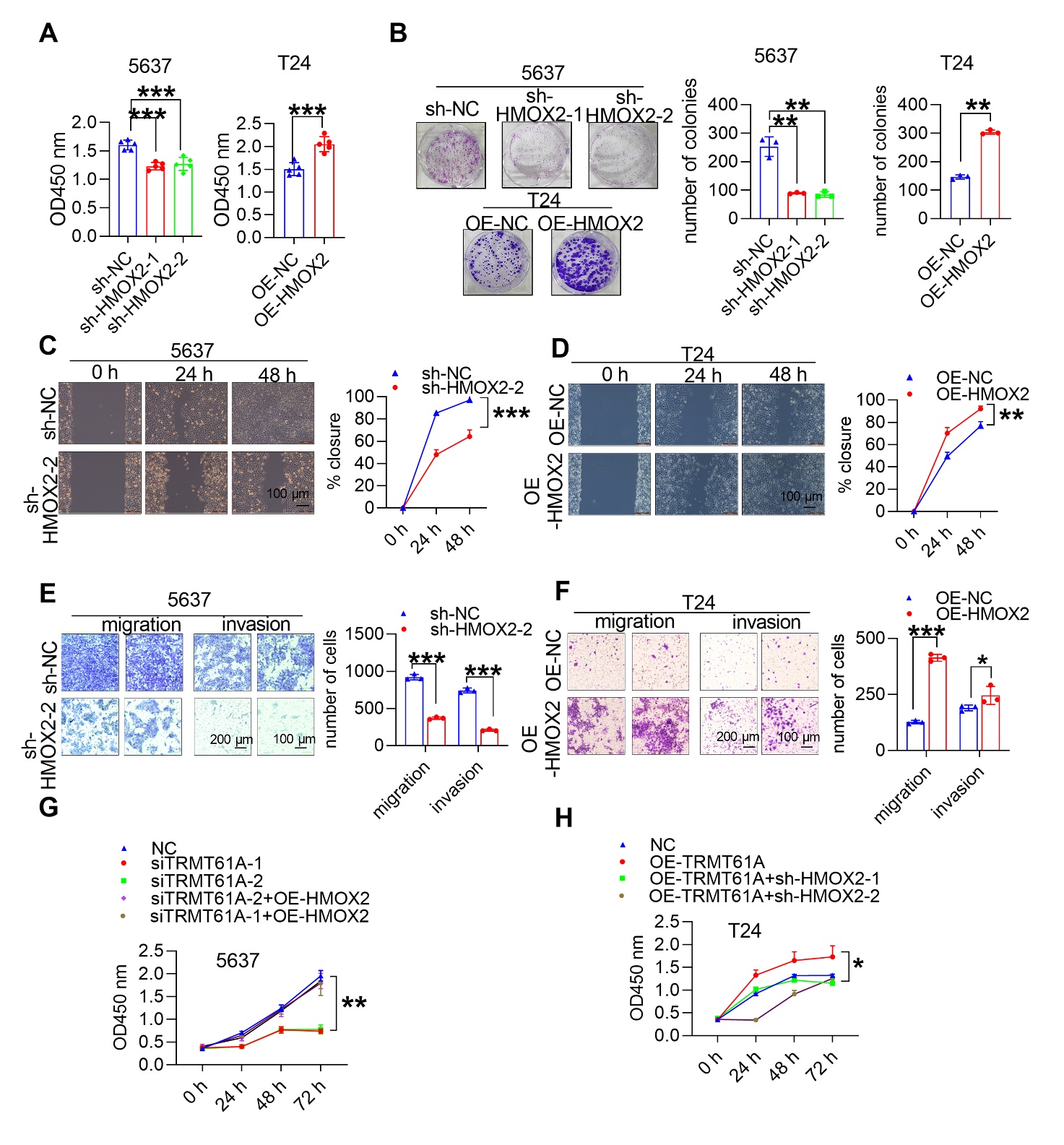


**Figure S4. HMOX2 is required for TRMT61A-induced BLCA progression.** (**A-B**) CCK8 (**A**) and colony formation (**B**) assays were performed in sh-NC and sh-HMOX2 5637 cells as well as empty vector (OE-NC) or HMOX2 overexpression (OE-HMOX2) T24 cells for 48 hours (CCK8 assays) or 14 days (colony formation assays) respectively. (**C-D**) Wound healing assays were performed in sh-NC or sh-HMOX2 5637 cells (**C**) as well as empty vector or HMOX2 overexpression T24 cells (**D**) 0, 24 and 48 hours after scratching. Scale bar: 100 μm. (**E-F**) Cell migration and invasion capabilities of sh-NC or sh-HMOX2 5637 cells (**E**) as well as empty vector or HMOX2 overexpression T24 cells (**F**) were measured by transwell migration and Matrigel invasion assays at 48 hours; and cells were fixed with crystal violet. Scale bar: 200 μm (left) and 100 μm (right). (**G**) 5637 cells were transfected with control siRNA (NC), si-TRMT61A, or combination of si-TRMT61A and OE-HMOX2 for 24, 48 or 72 hours, followed by CCK8 assays (OD450). (**H**) T24 cells were transfected with empty vector (NC), TRMT61A (OE-TRMT61A) expression construct, or combination of OE-TRMT61A and sh-HMOX2, followed by CCK8 assays (OD450) in functional rescue experiments. Two-tailed Student’s t-test was used for comparisons between two groups. Repeated measures ANOVA was used to analyze differences in cell viability among groups at different time intervals. ****P***<0.05, *****P***<0.01, and ******P***<0.001.

Supplementary Figure S5.


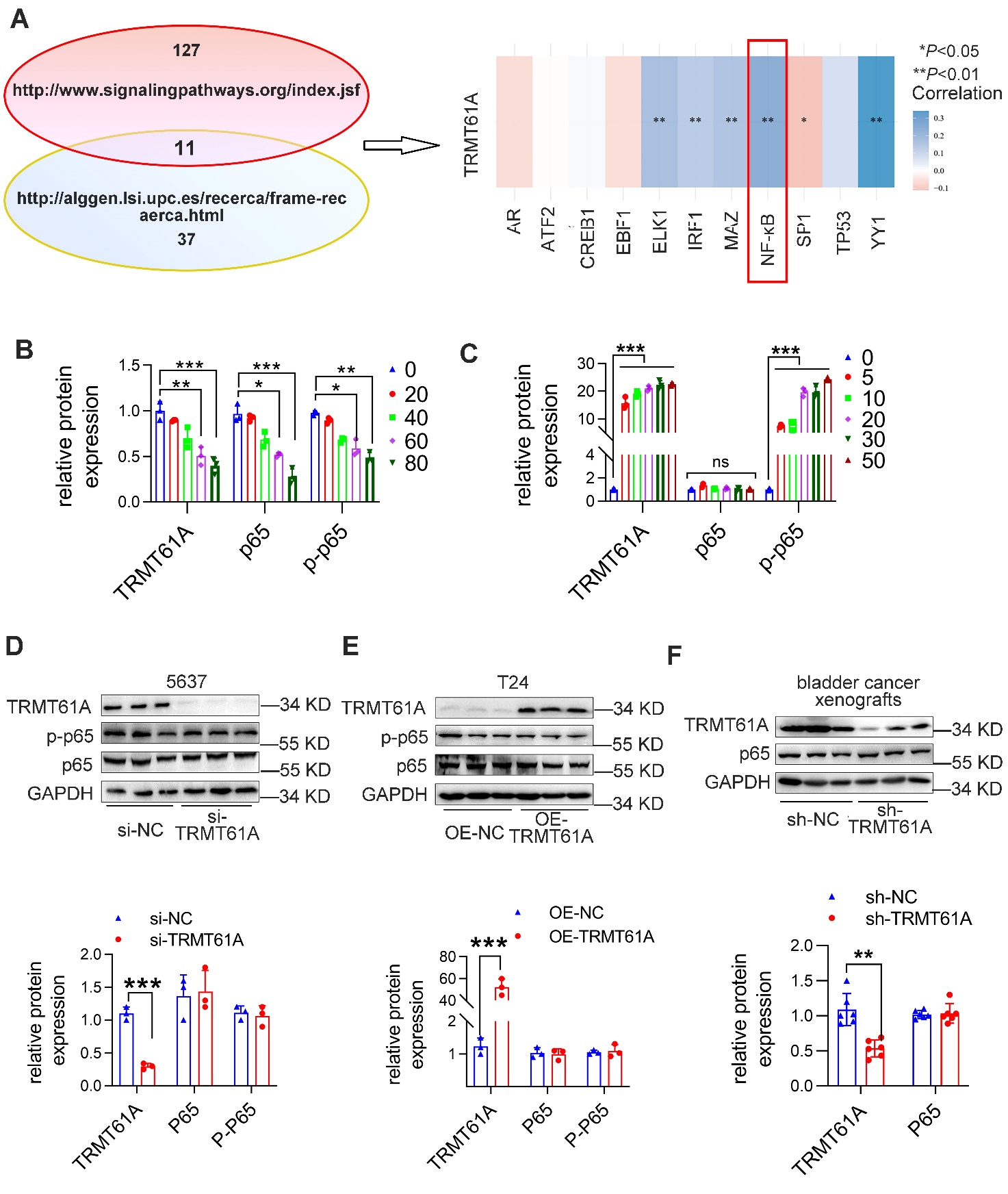


**Supplementary Figure S5. Nuclear transcription factor NF-κB is an upstream regulator of TRMT61A.** (**A**) Transcription factors that potentially bind to the TRMT61A promoter were predicted using http://www.signalingpathways.org/index.jsf and http://alggen.lsi.upc.es/recerca/frame-recerca.html online tools. Correlation analysis of the expression of the 11 TRMT61A promoter-binding nuclear transcription factors and TRMT61A in BLCA tissues in the TCGA database (bladder cancer database). (**B-C**) The statistical chart of 5637 cells were treated with SN50 for 24 hours (B) and T24 cells were treated with TNF-α for 24 hours (C), followed by western blot analysis of p65, pp65, and TRMT61A protein expression and quantification. (**D-E**) 5637 (D) and T24 (E) cells were transfected with si-NC, si-TRMT61A, or OE-NC, OE-TRMT61A for 48 h, followed by western blot analysis of TRMT61A, p65, and p-p65 protein expression and quantification. (**F**) Western blot analysis was performed to examine TRMT61A and p65 protein expression in tumor tissues from mice xenografted with negative control shRNA (sh-NC) or TRMT61A shRNA (sh-TRMT61A) 5637 cells, followed by quantification. GAPDH was used as the loading control. Two-tailed Student's t-test was used to compare differences between two groups, and ANOVA to compare differences among more than two groups. *P<0.05, **P<0.01, and ***P<0.001.

Supplementary Figure S6


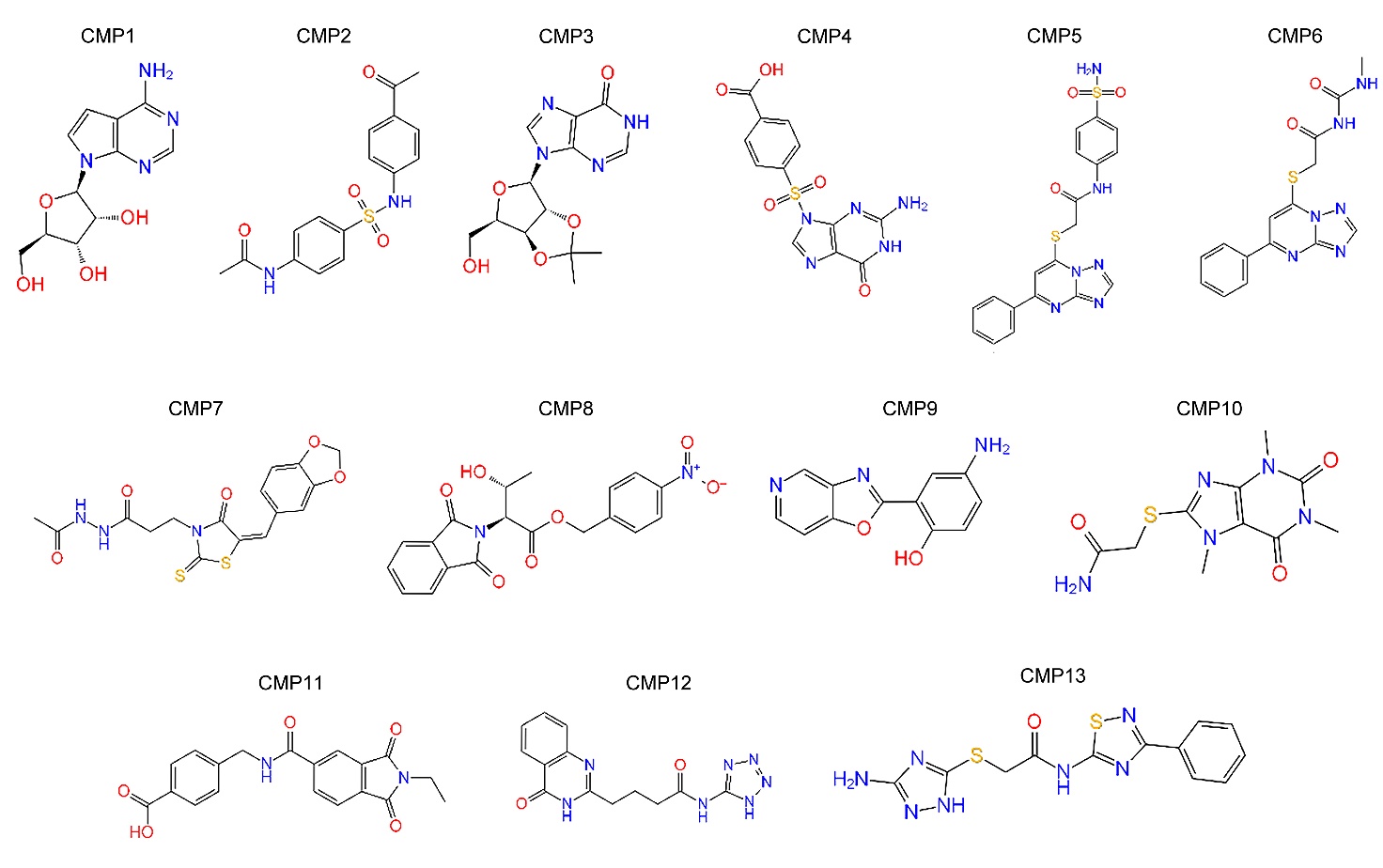


**Supplementary Figure S6.** **Chemical structures of small-molecule inhibitors of TRMT61A, identified by virtual screening of 210,331 small-molecule compounds in the Specs Library.**

Supplementary Figure S7.


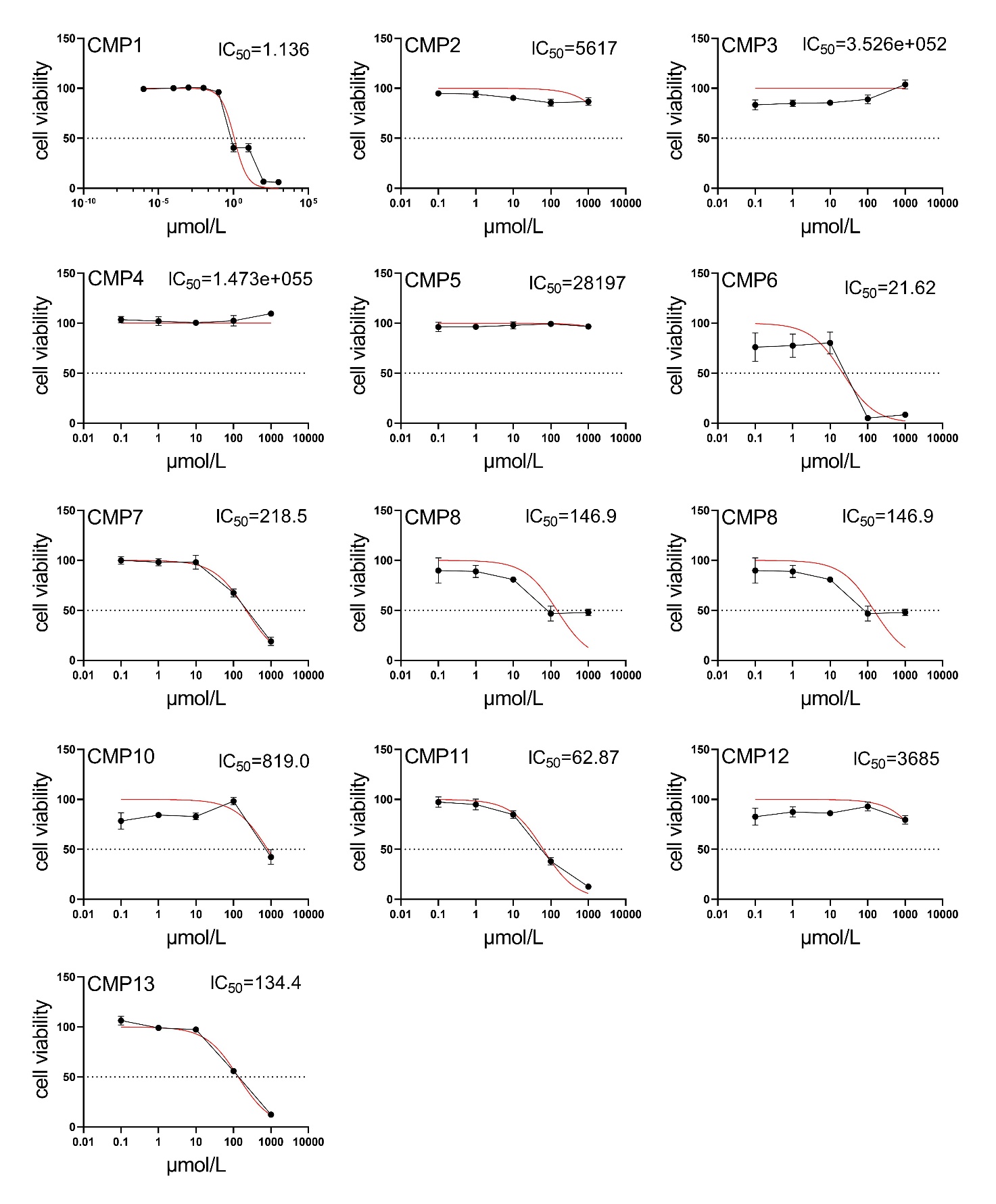


**Supplementary Figure S7.** **Effects of small-molecule TRMT61A inhibitors on 5637 BLCA cell proliferation and survival.** 5637 cells were treated with vehicle control or a series of concentrations of the 13 small molecular compound TRMT61A inhibitors, followed by CCK8 assays, and the half-maximal inhibitory concentration (IC_50_) values were determined. Error bars denote the SD for three biological repeats.

Supplementary Figure S8.


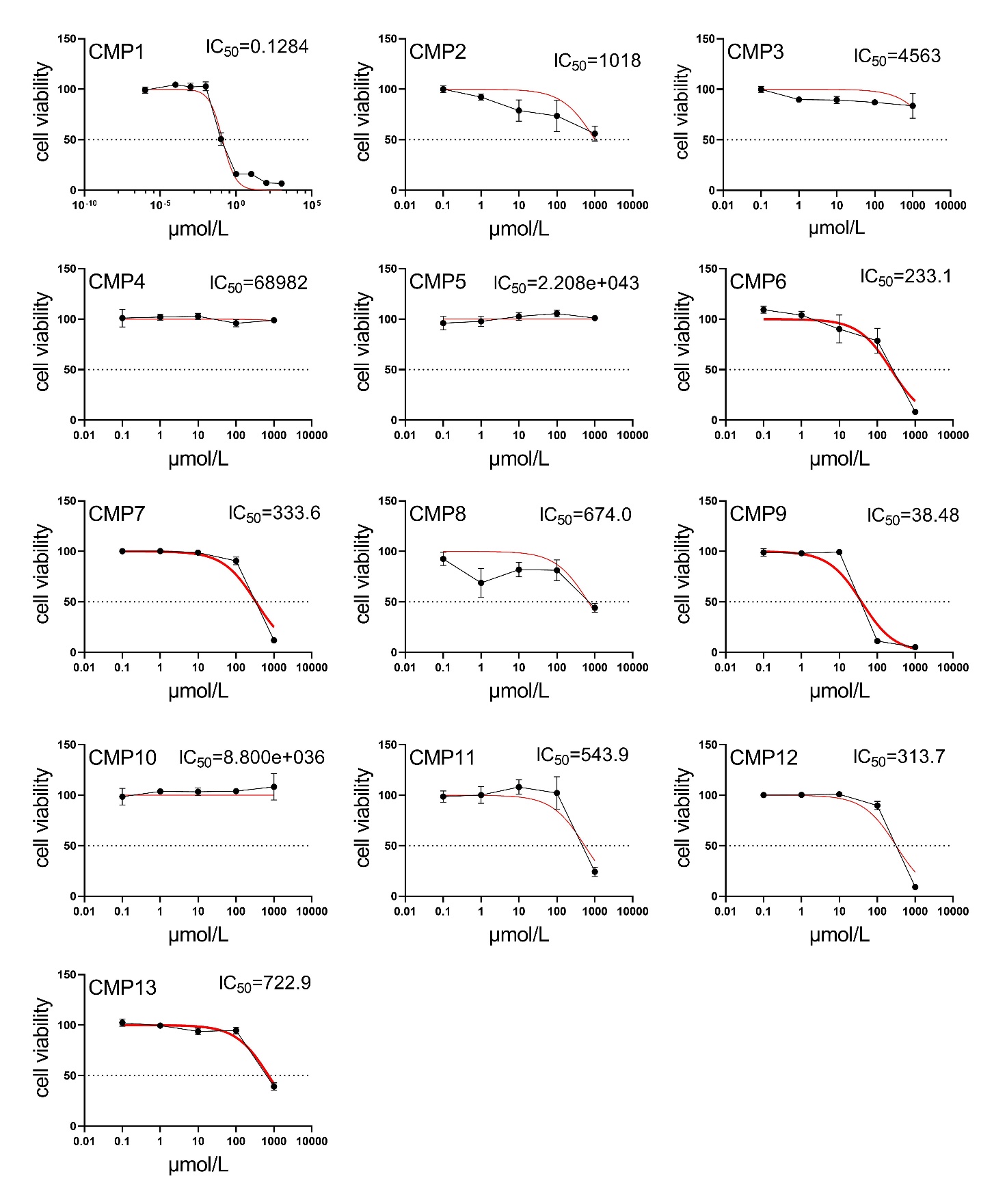


**Supplementary Figure S8. Effects of small-molecule TRMT61A inhibitors on T24 cell proliferation and survival.** T24 cells were treated with vehicle control or a series of concentrations of the 13 small molecular TRMT61A inhibitors, and the half-maximal inhibitory concentration (IC_50_) values were determined. Error bars denote the SD for three biological repeats.

**Reference**

1. O’Sullivan B, Brierley J, Byrd D, et al. The TNM classification of malignant tumours-towards common understanding and reasonable expectations. *Lancet Oncol*. 2017; 18: 849–851.

2. Chen IX, Newcomer K, Pauken KE, et al. A bilateral tumor model identifies transcriptional programs associated with patient response to immune checkpoint blockade. *PNAS*. 2020; 117(38): 23684-23694.

3. Chang Q, Chen P, Yin JJ, et al. Discovery and validation of bladder cancer related excreted nucleosides biomarkers by dilution approach in cell culture supernatant and urine using UHPLC-MS/MS. *J Proteomics*. 2023; 270: 104737.

4. Zhu YM, Jin L, Shi RH, et al. The long noncoding RNA glycoLINC assembles a lower glycolytic metabolon to promote glycolysis. *Mol Cell.* 2022; 82: 542-554.

5. Wang YY, Wang J, Li XY, et al. N-1-methyladenosine methylation in tRNA drives liver tumourigenesis by regulating cholesterol metabolism. *Nat Commun.* 2021; 12(1):6314.

6. Shen LS, Liang Z, Yu H. Dot Blot Analysis of N6-methyladenosine RNA Modification Levels. *Bio Protoc.* 2017; 7: e2095.

7. Jin HL, Ma JG, Xu JH, et al. Oncogenic role of MIR516A in human bladder cancer was mediated by its attenuating PHLPP2 expression and BECN1-dependent autophagy. *Autophagy.* 2021; 17: 840–854.

8. Wang RJ, Cao LX, Thorne RF, et al. LncRNA GIRGL drives CAPRIN1-mediated phase separation to suppress glutaminase-1 translation under glutamine deprivation. *Sci Adv.* 2021; 7: eabe5708.

9. Noel MO, Michael B, Craig AJ, et al. Geoffrey RH. Hutchison open babel: an open chemical toolbox. *J Cheminform*. 2011; 3: 33.

10. Oleg T, Arthur JO. AutoDock Vina: improving the speed and accuracy of docking with a new scoring function, efficient optimization, and multithreading. *J ComputChem.* 2010; 31: 455-461.
